# In Japan, individuals of higher social class engage in other-oriented humor

**DOI:** 10.1038/s41598-022-13755-4

**Published:** 2022-06-11

**Authors:** Ryota Tsukawaki, Tomoya Imura, Makoto Hirakawa

**Affiliations:** 1 Department of Social and Clinical Psychology, Hijiyama University, Hiroshima, Japan; 2 Graduate School of Teacher Education, Saga University, Saga, Japan; 3 Graduate School of Humanities and Social Sciences, Hiroshima University, Hiroshima, Japan

**Keywords:** Neuroscience, Psychology

## Abstract

Previous research on individual social class (SC) and humor has found support for the hypothesis that those with higher SC will engage in more dominant humor (aggressive humor) that derogates or degrades others. One rationale for introducing this hypothesis is the well-known theory that people with higher SC are more self-oriented; however, it has recently been shown that there may be cultural differences in this theory. In this study, using a Japanese sample objective measures (income and educational attainment) and subjective measures (perceived social status) and examined in relation to humor. Four types of humor assessed by the Humor Styles Questionnaire and two types of humor measured by the Dual Self-Directed Humor Scale were considered to investigate the relationship between SC and humor. Unlike prior findings obtained in Western countries, Study 1 (N = 344) and Study 2 (N = 604) consistently showed that SC and aggressive humor were unrelated. Rather, SC was shown to be positively associated with other-oriented humor in Japan, a country belonging to the Confucian cultural sphere of East Asia. The differences in results from these previous studies were discussed from a cultural contextual perspective.

## Introduction

### Social class and prosociality

Social class (SC) has a multifaceted structure consisting of both objective characteristics^1^, such as material wealth and access to resources, and subjective perception^2^ of one's position within the social hierarchy^3^. Since 2010, individuals with high SC are reportedly more self-oriented and have low levels of prosociality^4,5^. The most famous study was a series of experiments by Piff et al.^5^, who reported that individuals with high SC—measured through both subjective and objective measures such as income and education—were less likely to engage in a variety of prosocial behaviors. The study showed that individuals with high SC did not give points that would later be exchanged for money to anonymous partners in the dictator game, did not prefer to donate to charity, did not give points to assigned strangers in the trust game, and did not help experimental partners in need more than individuals with low SC.

Furthermore, the results showed that people with high SC were not only less likely to engage in prosocial behavior but also more likely to engage in a variety of unethical behaviors^6,7^. As a representative study, a series of experiments by Piff et al.^7^ revealed that people with high SC, as measured mainly by subjective indicators as well as objective indicators such as owning vehicles, were more likely to break the law while driving, steal valuable goods from others, lie in negotiations, commit fraud to increase the likelihood of winning prizes, and take unethical actions in the workplace (for example, increase sales by making personal long-distance calls at work or overcharging customers) than people with low SC. Other studies showed that people with a high SC, in contrast to those with a low SC, had a lower characteristic score for compassion^8^; inaccurate emotional reasoning related to others^9^; more behaviors that indicate a lack of attention to others, such as playing with one’s hands and doodling when interacting with strangers^10^; and a tendency to engage in humor to control others^11^. Some studies^12–14^ have not supported this high self-orientation of people with high SC, and the findings are mixed. Recently, Piff and Robinson^15^ discussed the need to identify psychological, ideological, and contextual factors that might explain such a gap.

The purpose of the current study was to examine the relationship between SC and humor using a Japanese sample. As discussed below, it was expected that the relationship between SC and humor would show a different pattern in Japan, a country belonging to the Confucian cultural sphere of East Asia, than in previous studies.

### Humor

Martin et al.^16^ conceptualized humor as a multidimensional structure with both adaptive and maladaptive components. The theory distinguishes between two adaptive types of humor (affiliative and self-enhancing humor) and two maladaptive types (aggressive and self-defeating humor). Those with high affiliative humor are more likely to be other-oriented and tell jokes to entertain others and develop interpersonal relationships, while those with high self-enhancing humor are more likely to maintain a humorous view of life even in adversity. People with high aggressive humor tend to use sarcasm and teasing frequently to dominate others, and people with high self-defeating humor tend to use humor directed at themselves excessively to gain approval from others. Martin et al.^16^ developed an instrument, the Humor Styles Questionnaire (HSQ), to distinguish and assess these four types of humor, and the HSQ is the most widely used instrument in research on individual differences in humor^17^.

Self-defeating humor has been conceptualized as maladaptive humor, but several studies have suggested an adaptive role for this humor. In a study conducted in Spain^18,19^, self-defeating humor was positively correlated with psychological wellbeing. Heintz and Ruch^20^ also found that the amount of humor generated in participants while responding to items on the self-defeating humor scale was associated with increased positive psychological outcomes. These results suggest that the HSQ's self-defeating humor scale may also have a mixed positive self-directed humor (SDH) component. Recently, Tsukawaki and Imura^21^ pointed out a problem with the self-defeating humor scale, a subscale of the HSQ. This scale measures individual differences in the use of SDH, but it focuses only on its inappropriate dimension. However, Tsukawaki and Imura^21^ argued that famous theorists of the past^22,23^ and contemporary researchers^24,25^ have emphasized the adaptive aspect of SDH. Adaptive SDH relates to humor to distance oneself from the problems one is facing and to downplay stressful events. This type of SDH can address fears and anxieties arising from problems in a positive way by allowing the individual to laugh at problems faced. Along these lines, Tsukawaki and Imura^21^ developed the Dual Self-Defeating Humor Scale (DSDHS), the first instrument to assess individual differences in SDH use from two dimensions, positive and negative. Initial validation of the DSDHS has provided promising evidence of reliability and validity^21,26^.

### SC and humor

It is believed that higher SC individuals tend to reinforce or maintain group hierarchy and status through the use of aggressive/dominant forms of humor^27^, and several studies have focused on this point. Qualitative studies on the use of humor by high- and low-status health care professionals^28,29^ suggest that high-status health care professionals are more likely to use humor to criticize or correct the errors of others. In addition, teasing studies have found that higher (vs. lower) status students tease their peers in a more aggressive manner, while lower (vs. higher) status students tease in a more prosocial manner^30^. A study examining the association between humor style and SC^11,31^ found that people with high SC tended to use more aggressive humor. Mendiburo-Seguel and Heintz^32^ also found that those with higher SC, as measured by educational attainment, scored higher on “hostile/dark” comic styles such as irony, satire, sarcasm, and cynicism. In sum, prior research has shown that people with high SC engage in dominant or hostile forms of humor due to increased self-oriented tendencies. In addition, no works showed SC differences in the styles of affiliative, self-enhancing, and self-defeating humor styles and in the remaining "lighter" comic styles.

Recently, it has been proposed that the meaning and function of SC varies across cultures, with the theory that high SC individuals have high levels of self-orientation regardless of culture but that high SC individuals from East Asian cultures have, in addition to this, high levels of other-orientation (see Miyamoto et al.^33^ for details). Due to a greater amount of resources and freedom associated with their acquired status, high-SC people have the tendency and ability to pursue the self and become more self-oriented. This tendency is common across cultures. However, Miyamoto et al.^33^ argued that at the same time, high-SC individuals are more likely than low-SC individuals to engage in the kinds of work that conform to and reaffirm the dominant view of self in their respective cultural contexts. Thus, in an individualistic cultural context such as the U.S., they would be expected to engage in tasks that promote the goals of uniqueness, competition, and the pursuit of personal success. On the other hand, in East Asian cultures, where Confucian teachings have shaped an interdependent view of self, high SC individuals would be expected to engage in work that promotes relationships and the interests of others. As a result, the high SC in East Asia is both self- and other-oriented. Indeed, Miyamoto et al.^33^ compared the association between SC and self- and other-orientation directly and cross-culturally from an analysis of a large international survey with a representative sample from Japan and the U.S. The results showed that across cultures, higher SC (as measured by subjective SC and educational attainment) was associated with higher self-orientation (such as self-esteem and goal attainment). In addition, in Japan, higher SC was associated with higher other-orientation, such as empathy and support for others, whereas in the U.S. the association was weaker.

Considering this cultural perspective, we expect that the relationship between SC and humor will show a different pattern in this study using an East Asian Japanese sample than in previous studies conducted in the U.S.^28–30^, Europe^11^, South America^32^, and West Asia^31^. That is, we expect that in addition to a positive association between SC and self-oriented humor (aggressive humor) that emphasizes hierarchical differences between self and others, we also expect that other-oriented humor and SC will be positively associated in the Japanese sample.

### Present study

The purpose of the current study was to test the association between SC and humor in a Japanese sample. Aggressive humor, a subscale of the HSQ, has a high underlying self-orientation, as it is a tendency to sarcastically and teasingly make fun of others to demonstrate one's superior social status. Thus, similar to previous studies^11,31^, we expected a positive association with SC (Hypothesis 1). Affiliative humor, the tendency to joke in order to entertain others and develop interpersonal relationships, is the humor that reflects the most other-orientation in the HSQ. Thus, we expected a positive association between affiliative humor and SC as well (Hypothesis 2). Self-enhancing humor, the tendency to maintain a humorous outlook even in stressful and adverse situations, appears to be unrelated to self- or other-orientation. However, previous studies have shown that self-enhancing humor is positively correlated with other-oriented measures such as empathy^34^, forgiveness^35^, and extraversion^33^. The relationship between self-enhancing humor and forgiveness was significant even after controlling for other humor styles. Consequently, we hypothesized a positive association between self-enhancing humor and SC (Hypothesis 3). Self-defeating humor is the tendency to seek approval from others through the use of overly self-sacrificing humor. At first glance, this humor may appear to be other-oriented, with interests and concerns directed toward others, but it is probably negatively motivated by fear of rejection by others. The evidence has not consistently shown an association between self-defeating humor and extraversion^36^. Thus, we hypothesized that self-defeating humor would be unrelated to SC (Hypothesis 4). Deleterious SDH in DSDHS, a concept derived from self-defeating humor, was also expected to be unrelated to SC for similar reasons (Hypothesis 5). Finally, benign SDH in the DSDHS was conceptualized as a tendency to laugh at the problems one faces and to make light of stressful events, but according to Tsukawaki and Imura^26^, this humor can also be used based on intrinsic benevolence and kindness toward others. In other words, such humor can convey, "I have the problem you have, but it is not so big that I cannot laugh this way." Therefore, we believe that benign SDH would also be positively correlated with SC, as it is positively correlated with extraversion and is other-oriented (Hypothesis 6).

In Study 1, we tested our hypotheses by measuring individual SC by an objective measure, household income. In Study 2, which was conducted to extend Study 1, SC was measured in a multidimensional way from the objective measures of household income and educational attainment and the subjective measure of one's perceived social status to test the hypotheses.

## Study 1

### Method

#### Participants and procedure

The participants in Study 1 consisted of 344 adults (209 males and 135 females), all of whom were Japanese. The mean age was 48.41 years (SD = 9.88), and the age range was 21–74 years. These participants were provided by Freeasy (https://freeasy.research-plus.net/lp/), a leading Japanese online research service operated by iBRIDGE Co., Ltd. Potential participants were notified of the questionnaire by e-mail from Freeasy. Of these people, those who provided informed consent responded via the internet in their free time.

#### Measures

All participants completed the HSQ (Martin et al.^16^; Japanese version by Yoshida^37^) and the DSDHS^21^ to assess individual differences in humor. In addition, the participants provided their household's annual after-tax income as an objective measure of SC.

The HSQ^16^ is a 32-item scale for assessing individual differences in four types of humor: affiliative, self-enhancing, aggressive, and self-defeating. The sample items are "I enjoy making people laugh" (affiliative), "If I am feeling depressed, I can usually cheer myself up with humor" (self-enhancing), "If I do not like someone, I often use humor or teasing to put them down" (aggressive), and "I will often get carried away in putting myself down if it makes my family or friends laugh" (self-defeating). Each subscale consists of 8 items and is rated on a 7-point Likert scale ranging from 1 (*totally disagree*) to 7 (*totally agree*). Cronbach's alpha values in this study ranged from 0.82 to 0.91.

The DSDHS^21^ is a scale for assessing individual differences in the two dimensions of SDH (deleterious and benign SDH). This is a 10-item scale with 5 items in each subscale. The sample items include the following: "I try to find a place for myself in my friendships by making a fool of myself to make my friends laugh" (deleterious SDH) and "I honestly accept problems I can’t solve and end up laughing about them with my family and friends" (benign SDH). A seven-point Likert scale ranging from 1 (*totally disagree*) to 7 (*totally agree*) was employed to rate the items. The Cronbach's alpha values in this study were 0.82 for "deleterious SDH" and 0.85 for "benign SDH".

Finally, household income, an objective measure of SC, was assessed in the following 15 categories: (a) less than 1 million yen, (b) 1–2 million yen, (c) 2–3 million yen, (d) 3–4 million yen, (e) 4–5 million yen, (f) 5–6 million yen, (g) 6–7 million yen, (h) 7–8 million yen, (i) 8–9 million yen, (j) 9–10 million yen, (k) 10–12 million yen, (l) 12–15 million yen, (m) 15–18 million yen, (n) 18–20 million yen, and (o) 20 million yen or more.

#### Data analysis

In the preliminary analysis, means and standard deviations were calculated for all study variables. In addition, a frequency analysis was conducted for household income, an objective measure of SC. Intercorrelations between humor and SC were calculated by zero-order correlational analyses. Finally, a hierarchical multiple regression analysis was performed to determine the predictive value of SC for each type of humor. In the first step of the hierarchical multiple regression analysis, age and gender (0 = male, 1 = female), coded as dummy variables, were included as control variables; in the second step, household income was added, and in the third step, an SC × gender interaction term was added. We also centralized the scores of these independent variables to avoid multicollinearity following Aiken and West^38^. We used HAD (statistical software running on Microsoft Excel; Shimizu^39^) to analyze our data.

#### Approval for human experiments

Ethical approval was obtained from the Ethical Committee of Graduate School of Education, Saga University for the study. All methods were carried out in accordance with relevant guidelines and regulations. All participants provided online informed consent prior to being monitored.

### Results and discussion

The means and standard deviations of the humor and SC measures are shown in Table 1. The distribution of participant income was as follows: less than 1 million yen (8.7%), 1–2 million yen (6.1%), 2–3 million yen (11.6%), 3–4 million yen (12.2%), 4–5 million yen (10.8%), 5–6 million yen (10.8%), 6–7 million yen (9.3%), 7–8 million yen (6.7%), 8–9 million yen (3.8%), 9–10 million yen (5.8%), 10–12 million yen (5.5%), 12–15 million yen (4.4%), 15–18 million yen (0.9%), 18–20 million yen (1.2%), and more than 20 million yen (2.3%). This distribution is roughly consistent with the distribution of household income in Japan conducted by the Japanese government^40^, and thus, the representativeness of the sample is considered to be somewhat guaranteed.

**Table 1:** Means, SDs, and zero-order correlations among study variables.

	1	2	3	4	5	6	7
1. AFH	–	0.30***	− 0.04	0.03	0.47***	− 0.19***	0.25***
2. SHE		–	0.22***	0.54***	0.63***	0.38***	0.16***
3. AGH			–	0.51***	0.13*	0.48***	0.05
4. SDH				–	0.42***	0.72***	0.07
5. BSDH					–	0.44***	0.18**
6. DSDH						–	− 0.01
7. Income							–
*M*	4.31	3.74	3.33	3.37	3.65	2.91	6.09
*SD*	0.99	0.90	0.92	1.07	1.28	1.34	3.53

*AFH* affiliative humor, *SHE* self-enhancing humor, *AGH* aggressive humor, *SDH* self-defeating humor, *DSDH* deleterious SDH, *BSDH* benign SDH, *M* mean, *SD* standard deviation.

*N* = 344.

*p < 0.05.

**p < 0.01.

***p < 0.001.

Table 1 shows that SC is significantly positively correlated with affiliative humor, self-enhancing humor, and benign SDH. No significant correlations were found between other types of humor and SC. Finally, as shown in Table 2, hierarchical multiple regression analysis revealed that SC was a significant predictor of affiliative humor, self-enhancing humor, and benign SDH, while aggressive humor, self-defeating humor, and deleterious SDH were not. Thus, the results did not support hypothesis 1 but did support hypotheses 2 through 5. These results indicate that, unlike in the West, SC is unrelated to aggressive humor in Japan. Rather, in Japan, which belongs to East Asia, SC was shown to be positively correlated with styles of humor that are thought to have an underlying other-orientation, such as affiliative humor, self-enhancing humor and benign SDH. Study 1 is limited, however, in that SC was measured by a single indicator, household income. In Study 2, we will measure SC in a more multidimensional way to see if similar results can be replicated.

**Table 2:** Hierarchical multiple regression analysis to predict six types of humor.

Predictor	Affiliative		Self-enhancing		Aggressive		Self-defeating		Deleterious SDH		Benign SDH	
	Δ*R*^2^	β	Δ*R*^2^	β	Δ*R*^2^	β	Δ*R*^2^	β	Δ*R*^2^	β	Δ*R*^2^	β
**Step 1**	0.01		0.01		0.05***		0.03*		0.03**		0.01	
Age		0.03		− 0.04		− 0.10		− 0.15**		− 0.10		0.01
Gender		0.07		− 0.08		− .024***		− 0.12*		− 0.19***		− 0.07
**Step 2**	0.07***		0.03**		0.00		0.01		0.00		0.04**	
Age		0.00		− 0.06		− 0.11		− 0.16**		− 0.10		− 0.01
Gender		0.08		− 0.07		− 0.24***		− 0.12*		− 0.19***		− 0.06
Income		0.26***		0.17**		0.04		0.08		− 0.01		0.18**
**Step 3**	0.00		0.00		0.00		0.01		0.00		0.00	
Age		0.00		− 0.06		− 0.11		− 0.16**		− 0.10		− 0.01
A: Gender		0.08		− 0.08		− 0.24***		− 0.13*		− 0.19***		− 0.06
B: Income		0.26***		0.16**		0.04		0.07		0.01		0.18**
A × B		0.02		− 0.04		− 0.04		0.06		0.02		0.01

*N* = 344.

*p < 0.05.

**p < 0.01.

***p < 0.001.

## Study 2

### Method

#### Participants and procedure

Study 2 included 607 adult Japanese participants (340 males and 267 females). The mean age was 49.53 years (SD = 12.23), and the age range was 22–91 years. These participants were recruited into the study in the same manner as in Study 1.

#### Measures

As in Study 1, all participants completed the HSQ and DSDHS to assess individual differences in humor and selected household income from 15 categories as an objective measure of SC. Cronbach's alphas on each humor scale were adequate in Study 2 (*α*s > 0.82). In addition, in Study 2, the participants also reported educational attainment, which was used as an objective measure of SC, and the MacArthur Scale of Subjective Socioeconomic Status^2^, which was used as a subjective measure.

Educational attainment was divided into six categories: (a) junior high school graduate, (b) high school graduate, (c) associate's degree (vocational/vocational training or academic), (d) bachelor's degree, (e) master's degree, and (f) doctoral degree.

We used the MacArthur Scale of Subjective Socioeconomic Status^2^, which we translated into Japanese. In this scale, the participants are presented with a graphic 10-step ladder representing ascending positions based on income, education level, and occupation and report their SC position in society in general by selecting a step on the ladder.

#### Data analysis

First, means and standard deviations were calculated for all study variables. Next, frequency analyses were conducted for household income and educational attainment, which are objective measures of SC. Intercorrelations between humor and SC were calculated by zero-order correlational analyses. Finally, a hierarchical multiple regression analysis was performed to determine the predictive value of each SC measure for each type of humor. Before conducting this analysis, each SC scale was centralized, and collinearity statistics were computed to ensure that the recommended values (VIFs < 5, Akinwande et al.^41^) were not exceeded (VIFs < 1.30). In the hierarchical multiple regression analysis, age and gender were included as control variables in the first step; in the second step, household income, education, and subjective SC were additionally entered as indicators of SC; in the third step, interaction terms between these indicators of SC and gender were added. As in Study 1, the values of these independent variables were centralized. For these analyses, HAD (Shimizu^39^) was used.

### Results and discussion

The means and standard deviations of the humor and SC measurements are shown in Table 2. The distribution of the participants' income was as follows: less than 1 million yen (5.9%), 1–2 million yen (8.6%), 2–3 million yen (11.2%), 3–4 million yen (15.7%), 4–5 million yen (11.2%), 5–6 million yen (10.5%), 6–7 million yen (8.6%), 7–8 million yen (7.9%), 8–9 million yen (3.6%), 9–10 million yen (4.4%), 10–12 million yen (4.6%), 12–15 million yen (3.5%), 15–18 million yen (1.6%), 18–20 million yen (0.3%), and more than 20 million yen (2.3%). As in Study 1, this distribution is roughly consistent with the distribution of household income in Japan. The distribution of educational attainment was as follows: junior high school graduate (2.5%), high school graduate (28.7%), associate's degree (20.4%), bachelor's degree (43.3%), master's degree (4.3%), and doctoral degree (0.8%).

In the zero-order correlation analysis shown in Table 3, household income and subjective SC were positively correlated with all types of adaptive humor (affiliative humor, self-enhancing humor, benign SDH). As shown in Table 4, hierarchical multiple regression analysis revealed that the subjective index of SC was a significant positive predictor of all other-oriented humor (affiliative humor, self-enhancing humor, and benign SDH). As shown in Table 4, hierarchical multiple regression analysis revealed that subjective indicators of SC were significant positive predictors of all other-oriented humor (affiliative humor, self-enhancing humor, and benign SDH). Household income, an objective measure of SC, significantly predicted only affiliative humor, while educational attainment was unrelated to all humor. The American Psychological Association^42^ argues that rather than integrating objective measures of SC, such as income and educational attainment, it is more useful to understand how each contributes to the outcome under study, and the data in this study support this view. In East Asian Confucian cultures, an other-oriented view of self is encouraged. The reason high SCs are more other-oriented is that the abundant resources they possess allow them to pursue it^33^. Since high income is related to control over resources^43^ and educational attainment is thought to be related to respect from others^44^, this study's finding that income predicts more other-oriented humor is not surprising.

**Table 3:** Means, SDs, and zero-order correlations among study variables.

	1	2	3	4	5	6	7	8	9
1. AFH	–	0.40***	0.10	0.25***	0.50***	− 0.02	0.25***	0.02	0.26***
2. SHE		–	0.10*	0.28***	0.56***	0.15***	0.14***	0.05	0.22***
3. AGH			–	0.47***	0.08*	0.42***	0.03	− 0.04	0.01
4. SDH				–	0.30***	0.68***	− 0.02	− 0.08	0.00
5. BSDH					–	0.25***	0.13**	0.01	0.18***
6. DSDH						–	− 0.02	− 0.06	− 0.09*
7. Income							–	0.23***	0.41***
8. Education								–	0.24***
9. SSC									–
*M*	4.50	3.64	3.00	3.06	3.70	2.45	5.92	4.21	4.59
*SD*	1.22	0.91	0.97	1.13	1.29	1.28	3.37	1.01	1.98

*N* = 607.

*AFH* affiliative humor, *SHE* self-enhancing humor, *AGH* aggressive humor, *SDH* self-defeating humor, *DSDH* deleterious SDH, *BSDH* benign SDH, *SSC* subjective social class, *M* mean, *SD* standard deviation.

*p < 0.05.

**p < 0.01.

***p < 0.001.

**Table 4:** Hierarchical multiple regression analysis to predict six types of humor.

Predictor	Affiliative		Self-enhancing		Aggressive		Self-defeating		Deleterious SDH		Benign SDH	
	Δ*R*^2^	β	Δ*R*^2^	β	Δ*R*^2^	β	Δ*R*^2^	β	Δ*R*^2^	β	Δ*R*^2^	β
**Step 1**	0.01		0.00		0.04***		0.01*		0.01*		0.00	
Age		0.01		0.01		− 0.15***		− 0.08		− 0.12**		− 0.01
Gender		0.10*		0.01		− 0.19***		0.05		− 0.05		0.04
**Step 2**	0.11***		0.05***		0.01		0.01		0.01		0.04***	
Age		0.03		0.02		− 0.15***		− 0.09*		− 0.12**		− 0.00
Gender		0.12**		0.02		− 0.20***		0.03		− 0.06		0.05
Income		0.19***		0.07		0.02		− 0.02		0.02		0.07
Education		− 0.06		− 0.01		− 0.08		− 0.08		− 0.05		− 0.04
SSC		0.19***		0.19***		0.02		0.03		− 0.09		0.16***
**Step 3**	0.01		0.02*		0.00		0.01		0.00		0.00	
Age		0.03		0.01		− 0.15***		− 0.09*		− 0.13**		− 0.00
A: Gender		0.12**		0.01		− 0.19***		0.03		− 0.06		0.04
B: Income		0.19***		0.10		0.02		− 0.01		0.03		0.07
C: Education		− 0.06		− 0.02		− 0.07		− 0.07		− 0.05		− 0.04
D: SSC		0.21***		0.19***		0.01		0.02		− 0.10*		0.16***
A × B		− 0.02		− 0.05		0.04		0.07		0.04		− 0.05
A × C		− 0.00		− 0.07		0.02		0.01		− 0.02		− 0.03
A × D		0.08		− 0.06		− 0.06		− 0.05		− 0.06		0.01

*N* = 607.

*SSC* subjective social class.

*p < 0.05.

**p < 0.01.

***p < 0.001.

In summary, Study 2 showed generally similar results to Study 1, finding that those with high SC (mainly by subjective measures) tended to use more other-oriented humor. The repeated finding of previous studies^11,31^ that high SC individuals engaged in aggressive humor was not indicated. Thus, as in Study 1, hypothesis 1 was not supported, but hypotheses 2 through 5 were supported.

## General discussion

The present study was the first to examine the relationship between subjective and objective SC using a Japanese sample and a wide range of measures of humor, a multidimensional construct. We expected that in Japan, a collectivist culture, the relationship between SC and humor would show a partially different pattern than in previous studies.

### SC and humor

The results of this study generally supported the prediction (Hypotheses 2, 3, and 6) that people with high SC would be more likely to engage in humor (affiliative humor, self-enhancing humor, benign SDH), which is thought to have an underlying other-orientation. These results differ from those obtained in previous studies conducted in the U.S.^28–30^, Europe^11^, South America^32^, and West Asia^31^ and are the first to be presented in the current study using a sample of Japanese from collectivist cultures. The theoretical model proposed by Miyamoto et al*.*^33^ assumes that in East Asia, where Confucian teachings have shaped an interdependent view of the self, high-SC people are more other-oriented, and the results presented in this study support this model. This is supported by the fact that affiliative humor, which is thought to reflect the most other-oriented trait, showed the strongest association with SC. Hypotheses 4 and 5 that self-defeating humor and deleterious SDH, which are expected to be weakly linked to other- and self-orientation, are not related to SC are also generally supported, except for the weak association between SC and deleterious SDH in Study 2.

Following the theory proposed by Miyamoto et al.^33^, in Japan, a collectivist culture, people with high SC would have both self- and other-orientation. Therefore, we assumed, as in previous studies, that SC and aggressive humor would be positively associated in the Japanese sample. However, throughout Studies 1 and 2, the data did not support Hypothesis 1, that high SC would be more likely to engage in aggressive humor. Self-orientation and other-orientation are conceptually distinct and coexist^45^, but only when the pursuit of one goal does not conflict with the pursuit of the other^33^. In other words, if the pursuit of one goal precludes the pursuit of the other, people must choose which goal to prioritize. An important point here is that when aggressive humor is used to emphasize the hierarchical difference between oneself and others or to try to dominate others, the goal of other-orientation, which is the goal of interpersonal harmony and development, is likely to be hindered. Kitayama^46^ argued that even in collectivist cultures, it is important to demonstrate individual independence, but this is only an option that can be chosen after satisfying interdependence with others. Thus, in Japan, when the goals of self- and other-orientation are in conflict, the other-orientation is given priority, which may be due to high-SC people not engaging in aggressive humor.

### Contribution to the literature

As noted in the introduction, there have been inconsistent results regarding the high self-orientation of high-SC people, pointing to the need to clarify the factors causing this gap^15^. Our contribution to the literature is that we extend the theoretical model proposed by Miyamoto et al.^33^ that high-SC people are more other-oriented in East Asia, which has a mutually cooperative view of self, to individual humor styles using a Japanese sample. Specifically, unlike previous studies conducted in the U.S.^28–30^, Europe^11^, South America^32^, and West Asia^31^, we found that Japanese high-SC people engage in other-oriented humor (affiliative humor, self-enhancing humor, benign SDH). Future research should examine whether different cultural contexts are indicated and support Miyamoto et al.’s^33^ theoretical model in factors such as emotional reasoning about others^9^ and lack of attention to others^10^.

### Limitations

There are several limitations to our study. The nonexperimental methodology used in the current study does not allow us to infer the direction of causality between constructs. In the regression equation, humor is predicted by SC, but this does not specify a causal relationship. In other words, we cannot rule out the possibility that SC is determined by an individual's use of humor. Alternative methodological approaches, such as longitudinal and experimental designs, should be implemented to uncover potential causal relationships between these constructs. It should also be noted that this study measures individual humor based solely on self-reported data. In general, adaptive humor is perceived as a desirable trait^27^, so social desirability may influence assessment. Therefore, it is useful to assign numerical scores of humor to individuals using peer assessment, observation, etc., and to confirm the reproducibility of the results.

### Summary

Recently, it has been proposed that the meaning and function of SC varies across cultures, with the theory that high SC is associated with a high level of self-orientation across cultures but that high SC in East Asian cultures has also been associated with a high level of other-orientation^33^. In the present study, we examined the relationship between SC and personal humor style using a Japanese sample from an East Asian culture. The results showed that high SC engaged in other-oriented humor (affiliative humor, self-enhancing humor, benign SDH). These results support the theoretical model proposed by Miyamoto et al.^33^, suggesting that the association between SC and self- and other-orientation may vary across cultures. Future research should test the validity of this theoretical model using a variety of psychological outcomes associated with self-orientation and other-orientation.

## Data Availability

The data that support the findings of this study are openly available in Mendeley Data at https://doi.org/10.17632/v62t9z83xb.1.
